# The Imbalance of B-Lymphocyte Subsets in Subjects with Different Glucose Tolerance: Relationship with Metabolic Parameter and Disease Status

**DOI:** 10.1155/2017/5052812

**Published:** 2017-04-16

**Authors:** Chao Deng, Yufei Xiang, Tingting Tan, Zhihui Ren, Chuqing Cao, Bingwen Liu, Gan Huang, Xiangbing Wang, Zhiguang Zhou

**Affiliations:** ^1^Department of Metabolism & Endocrinology, The Second Xiangya Hospital, Central South University, Changsha, Hunan 410011, China; ^2^Key Laboratory of Diabetes Immunology, Central South University, Ministry of Education, National Clinical Research Center for Metabolic Diseases, Changsha, Hunan 410011, China; ^3^Division of Endocrinology, Department of Medicine, Rutgers Robert Wood Johnson Medical School, New Brunswick, NJ, USA

## Abstract

B lymphocytes are involved in inflammation and are related to insulin resistance in obesity and type 2 diabetes (T2D). This study investigated the phenotype and frequency of B-lymphocyte subsets in subjects recently diagnosed with T2D (*n* = 60), impaired glucose regulation (IGR, *n* = 73), and normal glucose tolerance (NGT, *n* = 169) by flow cytometry. T2D subjects had an increased percentage of CD19^+^CD23^+^ (B-2) cells and a decreased percentage of CD19^+^CD23^−^ (B-1) cells attributing to CD19^+^CD23^−^CD5^−^ (B-1b) cells, but not CD19^+^CD23^−^CD5^+^ (B-1a) cells, compared to NGT and IGR subjects. The proportion of CD19^+^CD5^+^CD1d^hi^ (B10) cells did not differ between the IGR or T2D group and NGT controls. Of note, HbA1c and triglyceride showed a positive correlation with B-2 cells but an inverse correlation with B-1 and B-1b cells, which were independently associated with the presence of T2D by logistic regression models. In summary, this study shows an unbalanced proinflammatory phenotype of B-cell subsets correlated with glycemia and lipidemia in patients with T2D. Our data provide new insight into chronic activation of the immune system and subclinical inflammation in T2D. Further prospective studies are warranted to confirm our observations.

## 1. Introduction

The natural history of diabetes is characterized by a progressive deterioration of glucose metabolism status from euglycemia through prediabetes to type 2 diabetes (T2D) [1]. Obesity, insulin resistance, and insulin secretory dysfunction play different roles during each stage of T2D progression [2, 3]. Nutrition, physical activity, and genetics also influence the progression to type 2 diabetes. Chronic systemic inflammation is an important link between obesity, insulin resistance, and T2D [4]. Elevated proinflammatory cytokines are linked with decreased insulin sensitivity, while anti-inflammatory cytokine expressions are associated with better glucose status [4–7]. Components of the immune system including macrophages, T cells, neutrophils, and eosinophils have been implicated in adipose tissue inflammation and insulin resistance [8–11]. Recent findings showed that altered immune reactivity accompanies and proceeds in T2D [12, 13]. Accumulating evidence demonstrated that B lymphocytes are recruited to adipose tissue in diet-induced obese (DIO) mice and facilitate insulin resistance through proinflammatory T cells and production of pathogenic IgG antibodies [14]. The activation of B cells is also enhanced in patients with T2D [15]. Furthermore, B cells support T cell-mediated inflammation in subjects with obesity and T2D [16].

B cells can be divided into two subsets, B-1 and B-2 cells; B-1 cells can be further classified as B-1a and B-1b cells [17, 18]. It remains controversial about the role of B-cell subsets in obesity and T2D. A recent study demonstrated that B-1a cells attenuate insulin resistance and glucose metabolism through anti-inflammatory cytokine interleukin- (IL-) 10 and polyclonal IgM-dependent mechanisms, while B-2 cells exacerbate metabolic disease [19]. Harmon et al. reported that B-1b cells protect against obesity-associated inflammation and glucose intolerance through IgM mechanism in DIO mice [20]. IL-10-producing B (B10) cells, namely, regulatory B cells, control T-cell responses and suppress inflammation via IL-10 [21, 22]. Depletion of B-cell-specific IL-10 deteriorated adipose tissue inflammation and insulin resistance in DIO mice, whereas adoptive transfusion of adipose tissue IL-10-producing regulatory B cells improved those effects [23].

A recent study showed distinct immune cell phenotypes in T2D patients, which were associated with metabolic parameter [24]. Grossmann et al. reported that the immune and inflammatory profiles could be distinguished in each stage of T2D [25]. Of note, high white blood cell count predicts the development of T2D [26]. However, there is no study to investigate the role of B-cell subsets in the development and progression of T2D, especially in the early preclinical stage of disease. In our current study, we compared B-cell compartments in subjects with impaired glucose regulation (IGR) and T2D with those in normal glucose tolerance (NGT) subjects. We aimed to characterize the phenotype and frequency of B-lymphocyte subsets and their relationship with metabolic factors and disease status.

## 2. Materials and Methods

### 2.1. Participants

Parts of this study were presented as a poster at the 20th Annual Meeting of the Chinese Diabetes Society, Xiamen, China, 16–19 Nov 2016 [27]. Subjects with recently diagnosed T2D (*n* = 60) and IGR (*n* = 73) and age-, gender-, and BMI-matched controls with NGT (*n* = 169) (Table 1) were enrolled in the present study from the Second Xiangya Hospital of Central South University. The study was designed in accordance with the principle of the revised Helsinki protocol and approved by the Ethics Committees of the Second Xiangya Hospital of Central South University. All study participants gave written informed consent. Anthropometric and metabolic data were collected by experienced physicians.

Subjects underwent a standardized 75 g oral glucose tolerance test to investigate glucose status. Study participants were categorized according to WHO criteria, that is, fasting plasma glucose (FPG) < 6.1 mmol/L and 2 hour plasma glucose < 7.8 mmol/L as NGT and FPG ≥ 6.1 mmol/L or 2 hour plasma glucose ≥ 7.8 mmol/L as IGR or prediabetes. Inclusive criteria of T2D were as follows: (1) with a typical history of hyperglycemia; (2) negative for any islet autoantibodies (glutamic acid decarboxylase antibody (GADA), insulinoma-associated protein-2 antibody (IA-2A), and zinc transporter 8 antibody (ZnT8A)); and (3) disease duration < 1  year, and not on insulin treatment.

Exclusion criteria were (1) type 1 diabetes or secondary diabetes mellitus; (2) any malignant disease, autoimmune disease, inflammation, and infectious disease; (3) history of immunosuppressive medication or steroids for more than 7 days; and (4) pregnant women.

### 2.2. Laboratory Measurements

A fresh fasting venous blood sample was obtained for determination of glycemia, fasting C-peptide (FCP), total cholesterol (TC), triglycerides (TG), low-density lipoprotein cholesterol (LDL-C), and high-density lipoprotein cholesterol (HDL-C). FCP was detected by a chemiluminescence assay (Adiva Centaur System). HbA1c was assessed by high-performance liquid chromatography (Bio-Rad Laboratories). The islet autoantibodies were evaluated by radioligand assays as previously described [28].

### 2.3. Flow Cytometry

The peripheral blood mononuclear cells were isolated by standard Ficoll-Paque Plus density-gradient centrifugation, and the following monoclonal antibodies were used for human fluorescence-activated cell staining according to the manufacturer's instruction: APC-Cy7-conjugated anti-human CD19 (HIB19), PE-Cy7-conjugated anti-human CD5 (LT21), APC-conjugated anti-human CD1d (51.1), and PerCP-Cy5.5-conjugated anti-human CD23 (EBVCS-5), all from Biolegend. Cell acquisition was performed by using a BD Canto II system and analyzed with FlowJo software as described previously [28]. In brief, total B cells were gated in a SSC/CD19 dot plot. B-cell subsets were defined according to the surface marker expression as CD19^+^CD23^+^ (B-2), CD19^+^CD23^−^ (B-1), CD19^+^CD23^−^CD5^+^ (B-1a), CD19^+^CD23^−^CD5^−^ (B-1b), and CD19^+^CD5^+^ CD1d^hi^ (B10) cells.

### 2.4. Statistical Analysis

Data are presented as means ± standard deviation or as median values (25th–75th percentiles). Statistical analyses were performed using ANOVA or the nonparametric Kruskal-Wallis test for multiple group comparisons. The categorical variables were evaluated by *χ*^2^ tests. Spearman's correlations or Pearson's correlations were employed to evaluate associations of the percentages of B-cell subsets with clinical parameters. To assess the effects of B-cell subsets on the presence of IGR and T2D, multinomial logistic regression models were applied to adjust for confounding variables. Adjusted odds ratios (ORs) were provided with 95% CI and *P* value. Receiver operating characteristic (ROC) curves and the area under the curve (AUC) were calculated to evaluate performance of B-cell subsets in distinguishing T2D. SPSS 19.0 (IBM Corporation, Chicago, IL) and GraphPad Prism 5 (GraphPad Software, San Diego, CA) were used for data analysis. Two-tailed *P*  value < 0.05 was considered statistically significant.

## 3. Results

### 3.1. Anthropometric and Metabolic Characteristics

The baseline characteristics of NGT, IGR, and T2D subjects in groups are presented in Table 1. There were no significant differences in the age, gender, and BMI among the three groups. Patients with T2D had higher waist circumference (WC) and diastolic blood pressure than those of the NGT controls. The levels of waist-hip ratio (WHR), systolic blood pressure (SBP), and TG in subjects with T2D and IGR were elevated than those in NGT controls. There was a gradual increase in the FPG but a gradual decrease in the HDL-C level from NGT controls via individuals with IGR to T2D. The FCP level increased with IGR and remained stable despite progression to T2D.

### 3.2. Altered Phenotype of B-Cell Subsets

**Figure 1:**
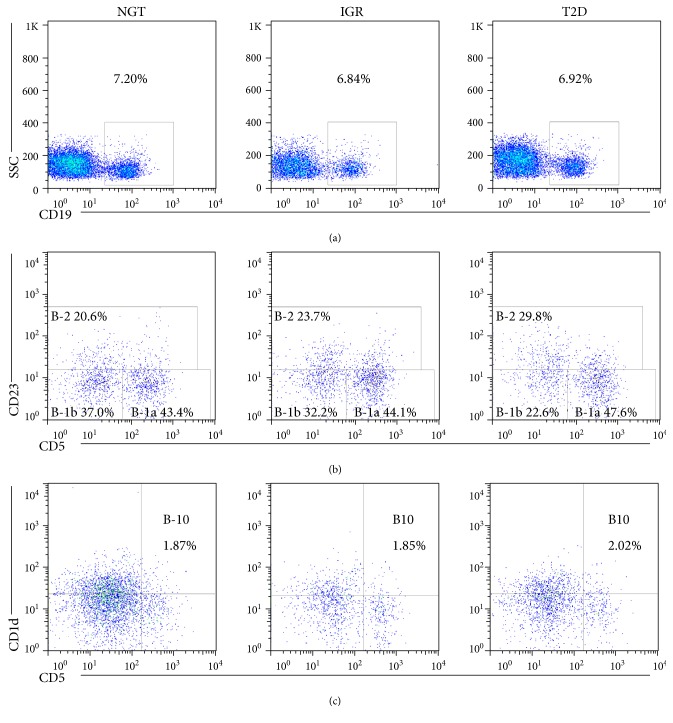
Representative flow cytometry analysis of peripheral B-cell subsets in subjects with NGT, IGR, and T2D. (a) Dots represent CD19^+^ B-cell frequency in total lymphocytes. Dead cells were excluded from the analysis based on their forward- and side-light scatter properties and propidium iodide staining. Doublets were excluded by FSC-A/FSC-H. (b) Representative dot plot showed the gating strategy for B-2 and B-1(B-1a, B-1b) cells in the CD19^+^ gate. (c) Representative dot plot showed the gating strategy for B10 cells gated on CD19^+^ B cells. IGR, impaired glucose regulation subjects; NGT, normal glucose tolerance subjects; T2D, type 2 diabetic subjects.

**Figure 2:**
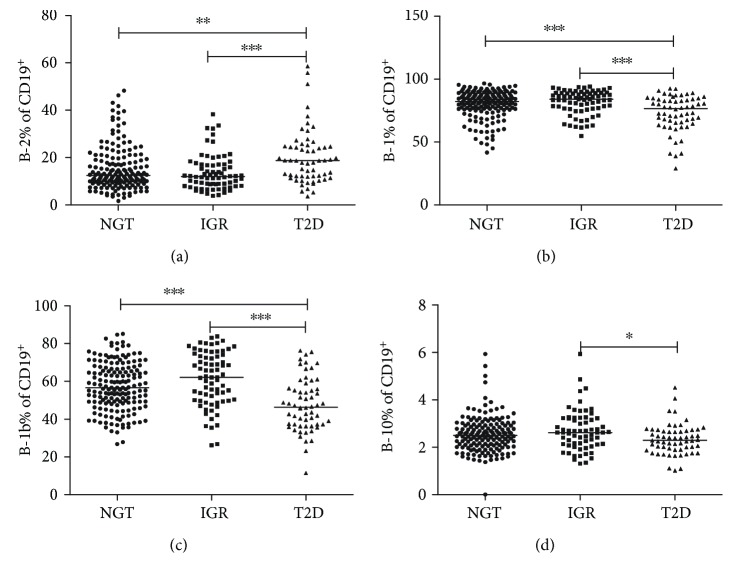
The frequency of B-cell subsets in subjects of different glucose metabolism status. The frequency of B-2 (a), B-1 (b), B-1b (c), and B10 (d) cells gated on CD19^+^ B cells. Each point represents the proportion of B-cell subsets of an individual. Horizontal lines show medians. ^∗^*P* < 0.05, ^∗∗^*P* < 0.01, and ^∗∗∗^*P* < 0.001.

Comparison of frequencies of B-cell subsets among NGT controls, subjects with IGR and T2D, are showed in Figures 1 and 2. There was no significant difference in the frequency of CD19^+^ and B-1a cells across different glucose status (*P* > 0.05 for both). Patients with T2D had higher B-2 cells (T2D versus NGT, *P* < 0.01; T2D versus IGR, *P* < 0.001) (Figure 2(a)), but T2D patients had lower B-1 cells compared with those of NGT and IGR subjects (*P* < 0.001 for both, Figure 2(b)). The proportion of B-1b cells tended to be increased in individuals with IGR (*P* = 0.084) and was decreased in patients with T2D (*P* < 0.001, Figure 2(c)) compared with those in the NGT group. B10 cells were comparable among individuals with NGT and IGR or T2D group and reduced from IGR to T2D (T2D versus NGT, IGR versus NGT, *P* > 0.05 for both; T2D versus IGR, *P* < 0.05) (Figure 2(d)). The frequencies of B-cell subtypes were comparable between IGR and NGT subjects and differed from IGR to T2D (Figure 2).

We also presented the percentages of B-cell subtypes in total PBMCs (see Supplementary Figure 1 available online at https://doi.org/10.1155/2017/5052812). Our results presented above remained the same for the percentages of CD19^+^, B-2, B-1, B-1a, and B-1b cells among different glucose tolerance groups. However, the percentage of B10 cells did not differ between groups.

### 3.3. B-Cell Subsets Associated with Metabolic Parameter and Disease Status

The correlations between B-cell subsets and clinical parameters regardless of glucose status are summarized in Table 2. Total B cells associated positively with LDL-C and negatively with HDL-C and FCP. The percentage of B-2 cells was negatively correlated, and the percentage of B-1 cells was positively correlated with female. Of note, HbA1c and TG exhibited a positive correlation with B-2 cells but an inverse correlation with B-1 and B-1b cells.

Given that association between gender with B-1 and B-2 cells, we compared the B-cell subsets between male and female in different glucose tolerance groups (Supplementary Figure 2). We found that the proportion of B-1 cells was significantly higher among females than among males in subjects with the NGT and IGR groups, but not with the T2D group. Other B-cell subsets were comparable between male and female in each group. Besides, we also examined the correlations between B-cell subsets and anthropometric data in different glucose tolerance status. In NGT group, the proportion of CD19^+^ B cells associated negatively with FCP (*r* = −0.159, *P* < 0.05) and positively with LDL-C (*r* = 0.172, *P* < 0.05). BMI showed a positive correlation with B-2 cells (*r* = 0.187, *P* < 0.05) but a negative correlation with B-1 cells (*r* = −0.141, *P* < 0.05). The B-1b cells exhibited a negative correlation with HbA1c (*r* = −0.164, *P* < 0.01). In the IGR group, B-1b cells positively correlated with FCP (*r* = 0.253, *P* < 0.05) and B-2 cells positively correlated with TG (*r* = 0.140, *P* < 0.05). Lastly, in patients with T2D, the percentage of B-2 cells was positively associated with SBP (*r* = 0.492, *P* < 0.05), TG (*r* = 0.148, *P* < 0.05), and HbA1c (Table 2), whereas B-1 and B-1b cells showed a negative association with TG (*r* = −0.106, *P* < 0.05; *r* = −0.183, *P* < 0.05) and HbA1c (Table 2). We tried to evaluate the association between B-2 cells and insulin resistance by using the C-peptide HOMA model, which uses plasma C-peptide concentrations to reflect endogenous insulin secretion [29]. We found a positive correlation between B-2 cells and insulin resistance in subjects with T2D (*r* = 0.397, *P* < 0.01), whereas there was no association between B-2 cells and insulin resistance in the IGR group.

**Figure 3:**
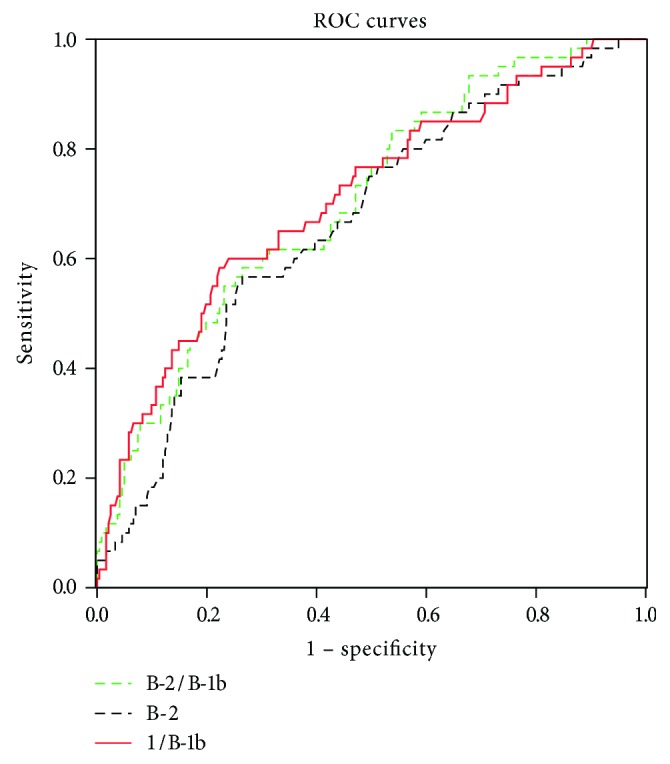
Receiver operating characteristic (ROC) curve analysis of performance of B-cell subsets in distinguishing type 2 diabetes.

Multinomial logistic regression analysis of correlations between B-cell subsets and glycemia status is shown in Table 3. The percentages of B-1 and B-1b cells were negatively correlated with T2D independent from WC, SBP, and TG, although there were no differences for CD19^+^ B cells and B-1a cells. Of note, B-2 cells showed a positive association with T2D but a negative correlation with IGR. Other B-cell subsets were not associated with IGR after adjustment for WC, SBP, and TG (*P* > 0.05). A further comparison between subjects with T2D and IGR was showed in Table 3. In addition, we plotted ROC curve analysis for discriminating T2D from the total study population (Figure 3). The AUC for B-2 and reciprocal B-1b cells was 0.667 (95% CI: 0.592–0.741) and 0.710 (95% CI: 0.635–0.785), respectively. Taking the imbalance of B-2 and B-1b cells into account, we further analyzed the AUC for B-2/B-1b and the result was 0.705 (95% CI: 0.633–0.776).

## 4. Discussion

A number of studies suggest that B cells are involved in the inflammatory reaction and correlated with insulin resistance in obesity and T2D. To our best knowledge, we for the first time characterize circulating B-cell compartments in subjects with different glucose tolerance status. Our data showed an unbalanced proinflammatory phenotype of B-cell subsets in patients with T2D and their correlation with metabolic characteristics and disease status.

B cells promote systemic inflammation in DIO mice or/and T2D patients by supporting proinflammatory T-cell function and secretion of IgG antibodies [14, 16]. Our study found that individuals with T2D had higher B-2 cells compared with those of the NGT and IGR groups (Figure 2(a)) and that B-2 cells were positively correlated with HbA1c and TG (Table 2). B-2 cells from obese mice produce more proinflammatory cytokine and less IL-10 compared to lean controls [16]. Shen et al. [19] found that high-fat diet (HFD) induced a significant increase in the relative percentage of B-2 cells. Adoptive transfer of B-2 cells exacerbates insulin resistance, whereas depletion of B-2 cells ameliorates insulin sensitivity in obese mice. IgG antibodies are shown to initiate adipose tissue inflammation and enhance insulin resistance [14]. These IgG antibodies are resident in visceral adipose tissue and secreted by adoptive B-2 cells [30].

Conversely, B cells have also been reported to play important roles in inhibiting inflammation and insulin resistance by IL-10 or/and polyclonal IgM mechanism [19, 20, 23]. Patients with T2D displayed a reduction in B-1 cells compared with those of the NGT and IGR groups, attributable to a reduction in B-1b cells (Figures 2(b) and 2(c)), which both expressed a negative association with HbA1c and TG (Table 2). B-1-derived IgM antibodies can alleviate inflammation and promote apoptotic cell clearance [31], which is impaired during the progress to obesity [32]. B-1a and B-1b cells reveal a unique function under different immunostimulation to *S. pneumoniae* [18]. The protective role of B-1 subsets against the inflammatory and metabolic abnormity of DIO mice remains controversial [19, 20]. HFD induces a significant decrease in the proportion of B-1a cells in the visceral adipose tissue. Remarkably, B-1a-cell transfusion protects against insulin resistance through IL-10 and polyclonal IgM mechanism [19]. Nevertheless, B-1b cells instead of B-1a cells are more frequent in Id3^Bcell KO^ mice and improve adipose tissue inflammation and glucose intolerance in DIO mice by IgM antibodies [20]. In addition, B-1-like cells are identified and accumulated in human omental adipose tissue, and IgM antibodies are negatively associated with inflammation and insulin resistance [20]. Taken together, similar to Th1/Th2 imbalance, the imbalance of B-2 and B-1b cells in total CD19^+^ B cells might point to an upregulated proinflammatory status with higher glycemia and lipidemia. A prospective and longitudinal study is warranted to confirm our observations.

We reported unique B10 cells in patients with autoimmune diabetes in our previous publication [28]. In our current study, we did not find any differences in B10 cells between the NGT controls and IGR or T2D group (Figure 2(d)). B10 cells have a regulatory function in inflammation, infection, and autoimmunity [21, 22, 33]. Loss- and gain-function study of B10 cells showed the protective role in adipose tissue inflammation [23]. Several phenotypic B-cell subsets are capable of producing IL-10 upon stimulation and of suppressing inflammatory processes in human [34, 35] but lack the suppressive capacity in systemic lupus erythematosus patients [34, 35]. Whether the regulatory function of IL-10-producing B cells in T2D is impaired requires further confirmatory experiments in the future study.

We were unable to determine any difference in B-cell subsets between NGT and IGR subjects (Figure 2), which suggested that obesity-related inflammation and insulin resistance might play a more important role in the change of B-cell subsets compared with hyperglycemia, given that the IGR and T2D subjects enrolled in the study were overweight, but not obese. This is in line with previous results that obesity has a substantial effect on the lifetime risk of prediabetes and diabetes [1] and induces a phenotypic switch in adipose tissue macrophage polarization [9]. The percentage of visceral adiposity and the risk factors for cardiovascular diseases are generally higher among Asian individuals compared with white populations with the same BMI or waist circumference. The metabolically obese phenotype of patients who have normal BMI level but increased visceral adiposity has been correlated with high risk of developing T2D [36]. Asians are more genetically susceptible to insulin resistance and diabetes than Western counterparts [36]. Besides, among Asian young patients with early-onset diabetes (age at diagnosis ≤ 40  years), nearly 40% had a lean, nonautoimmune phenotype with low levels of *β*-cell function [37]. In addition, epigenetic factors including gestational or overt diabetes in pregnancy, low birth weight, exposure to undernutrition in utero, micronutrient imbalances, and gut microbiota might contribute to increased risk for T2D [38, 39].

In the multinomial logistic regression model, B-2 cell was negatively associated with IGR but positively correlated with T2D. In contrast to B-2 cells, B-1b cells decreased in T2D patients and associated negatively with the presence of T2D independent of SBP, WC, and TG. These might suggest vital roles of B-cell subset in the pathogenesis of T2D and support the concept that metabolic syndrome components, SBP, TG, WC, and hyperglycemia, together affect the immune system in T2D and that the central mechanism of metabolic syndrome is insulin resistance and inflammation. The potential value of B-cell subsets in the determination of T2D was evaluated by ROC analysis. However, the AUC assays did not yield good model performance (AUC = 0.667 ~ 0.710), which was likely due to comparatively large individual variation (Figure 2). Improved methodological aspects and integration with other parameter may optimize the diagnostic value.

Our study shows for the first time distinct frequencies of B-cell subsets in T2D. This provides new insight into the spectrum of immune and inflammatory profiles associated with human T2D [24, 25, 40]. However, this study has also some limitations as follows. This cross-sectional study does not allow us to draw a conclusion regarding a causal interrelation between diabetes, inflammation, and immunity. Besides, we do not address infiltrating immune cells from adipose tissue, serum cytokines, or immunoglobulin levels.

## 5. Conclusions

This study shows the unbalance of B-cell subsets toward proinflammatory phenotype in T2D patients, and the B-cell subsets are correlated with glycemia and lipidemia. Our data provide new insight into chronic activation of immune system and subclinical inflammation in T2D. Further prospective studies as well as in vitro function experiments are warranted to confirm our observations.

## Supplementary Material

Supplementary Figure 1. The percentage of B-cell subsets in subjects of different glucose metabolism status. Dots represent B-2 (A),B-1 (B), B-1b (C), B10 (D) cell frequencies in tatol lymphocytes. Data shown as scatter plots with medians ∗P<0.05, ∗∗P<0.01, ∗∗∗P<0.001. IGR, impaired glucose regulation subjects; NGT, normal glucose tolerance subjects; T2D, type 2 diabetic subjects. Supplementary Figure 2. The frequency of B-cell subsets in different gender group according to different glucose metabolism status. The frequency of B-2 (A),B-1 (B), B-1b (C), B10 (D) cells gated on CD19+ B cells. Error bars indicate means mean ± SD ∗P<0.05, ∗∗P<0.01. IGR, impaired glucose regulation subjects; NGT, normal glucose tolerance subjects; T2D, type 2 diabetic subjects.

## Figures and Tables

**Table 1:** Anthropometric and metabolic data of subjects according to glucose tolerance.

	NGT (*n* = 169)	IGR (*n* = 73)	T2D (*n* = 60)
Male (%)	40.2 (68/169)	38.4 (28/73)	46.7 (28/60)
Age (y)	46.38 ± 11.07	49.86 ± 11.07	47.75 ± 13.52
BMI (kg/m^2^)	23.79 ± 3.31	24.26 ± 2.92	24.33 ± 3.36
WC (cm)	82.23 ± 10.52	84.93 ± 9.01	85.48 ± 8.18^∗^
WHR	0.85 ± 0.06	0.87 ± 0.06^∗^	0.88 ± 0.05^∗∗^
SBP (mmHg)	116.09 ± 15.07	122.59 ± 16.90^∗^	128.50 ± 16.30^∗∗^
DBP (mmHg)	75.68 ± 11.12	78.80 ± 11.93	84.15 ± 9.37^∗∗^
TG (mmol/L)^§^	1.16 (0.78–1.78)	1.49 (1.08–2.06)^∗^	2.03 (0.89–2.97)^∗∗∗^
TC (mmol/L)	5.09 ± 0.96	5.03 ± 0.95	5.12 ± 1.16
LDL-C (mmol/L)	2.85 ± 0.86	3.12 ± 0.80	3.06 ± 0.82
HDL-C (mmol/L)	1.36 (1.11–1.59)	1.26 (1.09–1.46)^∗^	1.09 (0.80–1.31)^∗∗∗^^†^
HbA1c (%)	NA	NA	6.70 (5.60–8.50)
HbA1c (mmol/L)	NA	NA	50 (38–69)
FPG (mmol/L)^¶^	5.20 (4.80–5.50)	5.70 (5.20–6.20)^∗∗∗^	7.19 (5.71–8.79)^∗∗∗^^†††^
FCP (mmol/L)^¶^	0.45 (0.34–0.63)	0.55 (0.45–0.73)^∗∗^	0.64 (0.37–0.79)^∗^

Data are % (*n*), mean ± SD, and median (25th–75th percentile). ^§^Compared after Log transformation. ^¶^Compared by the nonparametric Kruskal-Wallis test; IGR: impaired glucose regulation subjects; N/A: not appropriate; NGT: normal glucose tolerance subjects; T2D: type 2 diabetic subjects; BMI: body mass index; DBP: diastolic blood pressure; FCP: fasting C-peptide; FPG: fasting plasma glucose; HbA1c: glycated hemoglobin; HDL-C: high-density lipoprotein cholesterol; LDL-C: low-density lipoprotein cholesterol; TC: total cholesterol; TG: triglycerides; SBP: systolic blood pressure; WC: waist circumference; WHR: waist-hip ratio; ^∗^*P* < 0.05 compared with that in the NGT, ^∗∗^*P* < 0.01 compared with that in the NGT, ^∗∗∗^*P* < 0.001 compared with that in the NGT, ^†^*P* < 0.05 compared with that in the IGR, and ^†††^*P* < 0.001 compared with that in the IGR.

**Table 2:** Association between peripheral B-cell subset percentages and anthropometric and metabolic parameter.

	CD19	B-2	B-1	B-1a	B-1b	B10
Sex^¶^	0.036	**−0.185** ^∗∗^	**0.156** ^∗∗^	0.185	−0.009	−0.045
Age	0.052	0.012	0.002	−0.032	0.072	−0.029
BMI	−0.016	0.041	−0.043	−0.118	0.094	0.031
WC	0.039	0.033	−0.057	−0.069	0.052	0.048
WHR	0.056	0.045	−0.051	−0.051	0.034	0.032
SBP	0.114	0.006	−0.046	0.013	−0.066	−0.121
DBP	0.060	0.114	−0.111	−0.051	−0.023	−0.063
TC	0.117	0.064	−0.028	−0.049	0.025	0.022
TG^¶^	0.054	**0.198** ^∗^	**−0.181** ^∗^	−0.006	**−0.183** ^∗∗^	0.007
LDL-C	**0.151** ^∗^	0.027	−0.023	−0.108	0.082	0.048
HDL-C	**−0.126** ^∗^	−0.071	0.088	0.076	0.046	−0.055
FPG^¶^	0.049	0.096	−0.107	0.038	−0.093	−0.061
HbA1c^¶§^	0.090	**0.185** ^∗∗^	**−0.204** ^∗∗^	0.093	**−0.327** ^∗∗∗^	−0.088
FCP^¶^	**−0.127** ^∗^	0.068	−0.035	−0.144	0.062	−0.047

Association analyses between peripheral B-cell subset percentages (after Log transformation) and anthropometric and metabolic parameter were performed using Pearson's test. ^¶^Spearman's correlations. ^§^This correlation was only present in T2D individuals. ^∗^*P* < 0.05, ^∗∗^*P* < 0.01, and ^∗∗∗^*P* < 0.001.

**Table 3:** Adjusted logistic regression analysis for the presence of T2D and IGR.

	T2D versus NGT		IGR versus NGT		T2D versus IGR	
	OR (95% CI)	*P* value	OR (95% CI)	*P* value	OR (95% CI)	*P* value
CD19	1.677 (0.892–3.154)	0.109	1.305 (0.772–2.205)	0.320	1.132 (0.539–2.380)	0.743
B-2	1.703 (1.189–3.261)	0.018	0.463 (0.306–0.701)	2.730 × 10^−4^	2.800 (1.398–5.607)	0.004
B-1	0.119 (0.017–0.844)	0.033	3.443 (0.965–13.756)	0.054	0.015 (0.001–0.239)	0.003
B-1b	0.075 (0.017–0.342)	0.001	3.222 (0.931–11.146)	0.065	0.052 (0.010–0.273)	4.794 × 10^−4^
B-1a	1.539 (0.826–2.867)	0.174	1.018 (0.628–1.652)	0.941	1.598 (0.733–3.486)	0.239
B10	0.421 (0.137–1.291)	0.130	1.436 (0.856–2.410)	0.170	0.357 (0.122–1.049)	0.061

The frequency of B-cell subsets has been Log transferred. Adjusted ORs are given with 95% CI and *P* value.
