# Supplementary material for: The Imbalance of B-Lymphocyte Subsets in Subjects with Different Glucose Tolerance: Relationship with Metabolic Parameter and Disease Status
Source: J Diabetes Res. 2017 Apr 16;2017:5052812. doi: 10.1155/2017/5052812 (PMC5410374; doi:10.1155/2017/5052812)
Supplement: Supplementary file 1 — Supplementary Figure 1. The percentage of B-cell subsets in subjects of different glucose metabolism status. Dots represent B-2 (A),B-1 (B), B-1b (C), B10 (D) cell frequencies in tatol lymphocytes. Data shown as scatter plots with medians ∗P<0.05, ∗∗P<0.01, ∗∗∗P<0.001. IGR, impaired glucose regulation subjects; NGT, normal glucose tolerance subjects; T2D, type 2 diabetic subjects. Supplementary Figure 2. The frequency of B-cell subsets in different gender group according to different glucose metabolism status. The frequency of B-2 (A),B-1 (B), B-1b (C), B10 (D) cells gated on CD19+ B cells. Error bars indicate means mean ± SD ∗P<0.05, ∗∗P<0.01. IGR, impaired glucose regulation subjects; NGT, normal glucose tolerance subjects; T2D, type 2 diabetic subjects. [file 5052812.f1.docx]

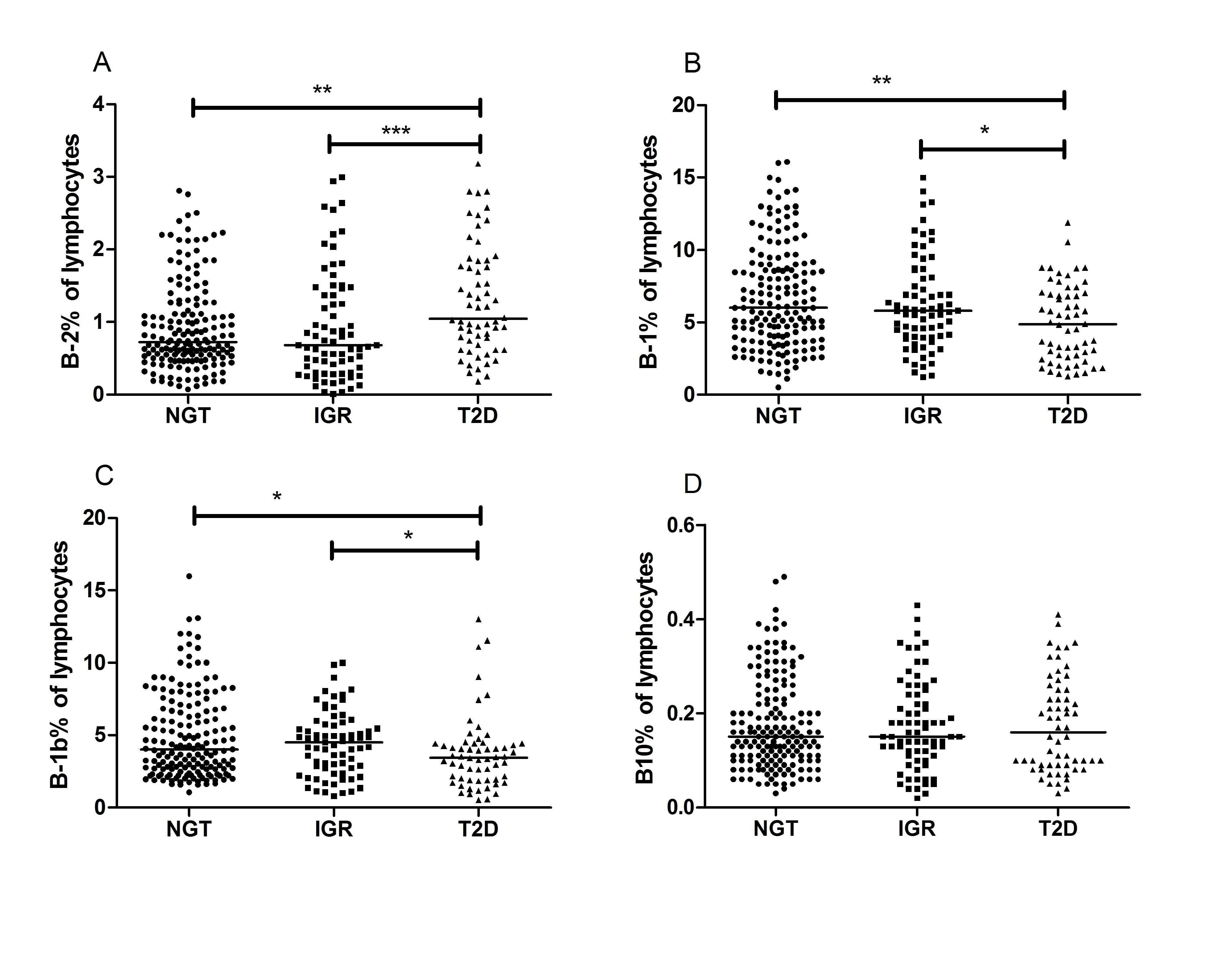


**Supplementary Figure 1**. The percentage of B-cell subsets in subjects of different glucose metabolism status. Dots represent B-2 (A),B-1 (B), B-1b (C), B10 (D) cell frequencies in tatol lymphocytes. Data shown as scatter plots with medians * *P* < 0.05, ** *P* < 0.01, *** *P* < 0.001. IGR, impaired glucose regulation subjects; NGT, normal glucose tolerance subjects; T2D, type 2 diabetic subjects.


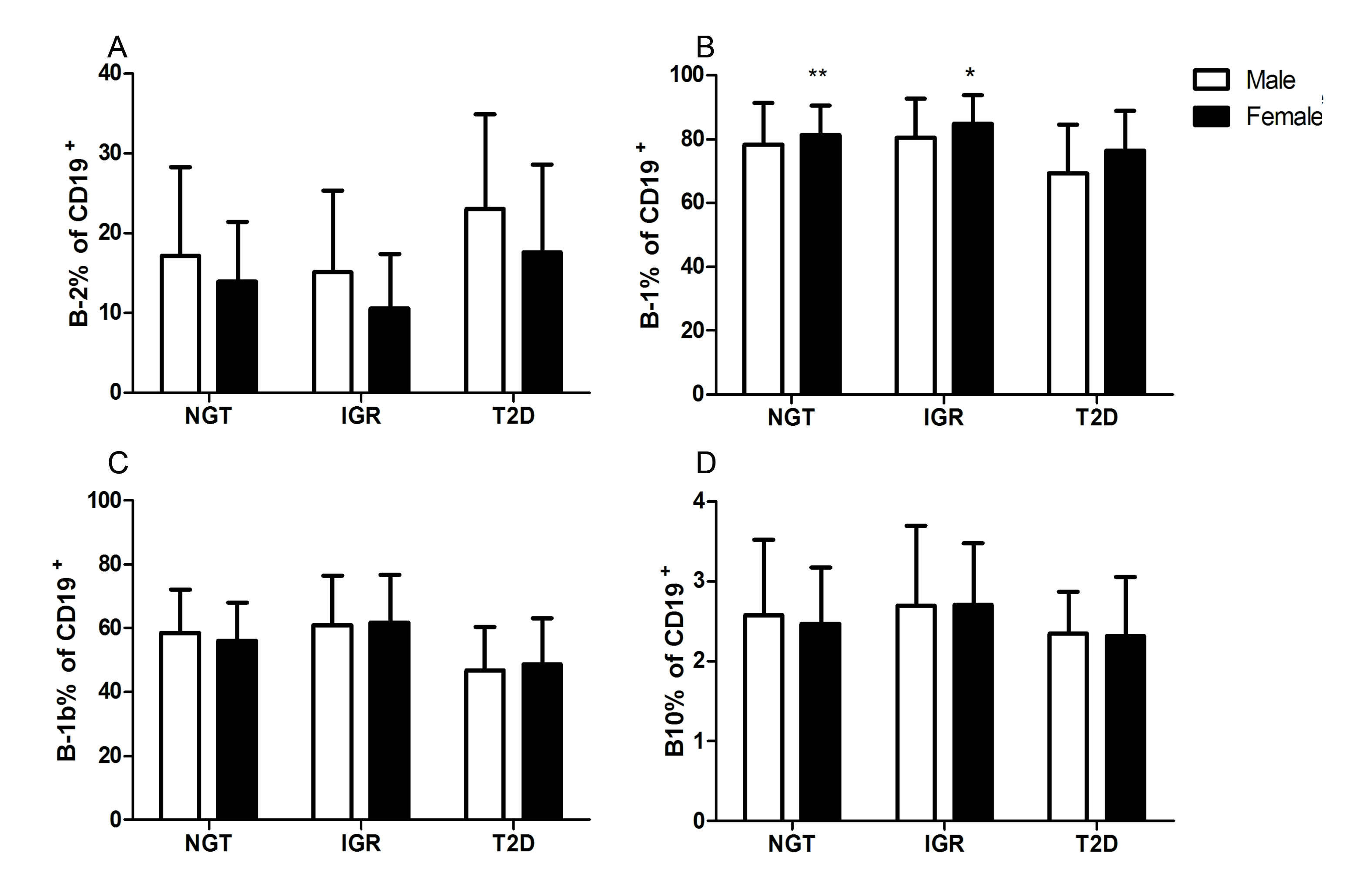


**Supplementary Figure 2**. The frequency of B-cell subsets in different gender group according to different glucose metabolism status. The frequency of B-2 (A),B-1 (B), B-1b (C), B10 (D) cells gated on CD19^+^ B cells. Error bars indicate means mean ± SD * *P* < 0.05, ** *P* < 0.01. IGR, impaired glucose regulation subjects; NGT, normal glucose tolerance subjects; T2D, type 2 diabetic subjects.
