# Supplementary figures and images for: The Imbalance of B-Lymphocyte Subsets in Subjects with Different Glucose Tolerance: Relationship with Metabolic Parameter and Disease Status
Source: J Diabetes Res. 2017 Apr 16;2017:5052812. doi: 10.1155/2017/5052812 (PMC5410374; doi:10.1155/2017/5052812)

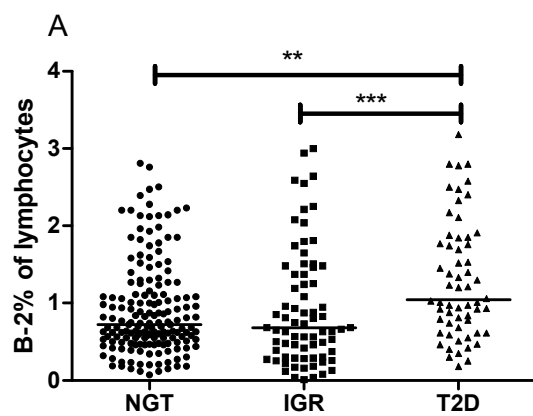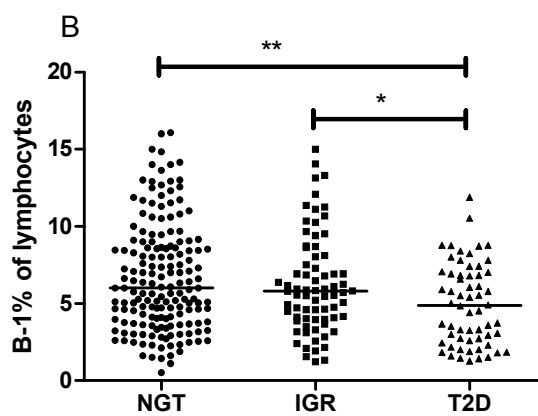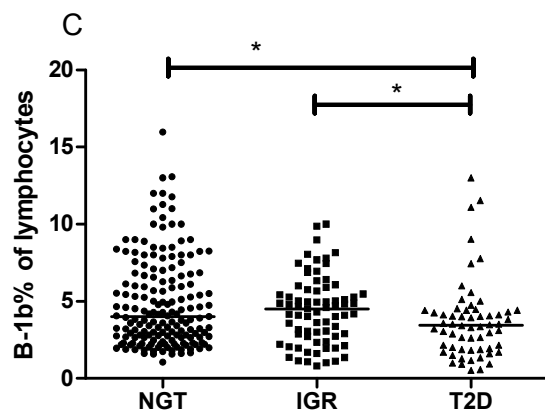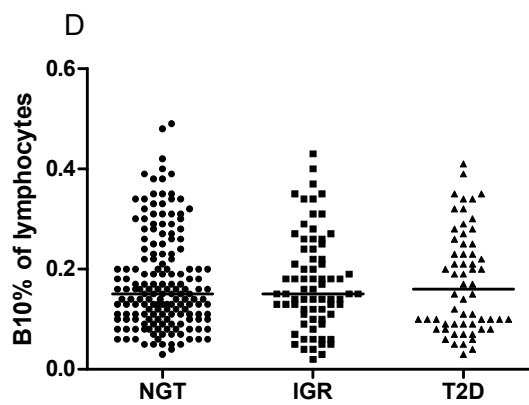

Supplement: Supplementary file 2 [file 5052812.f2.pdf]

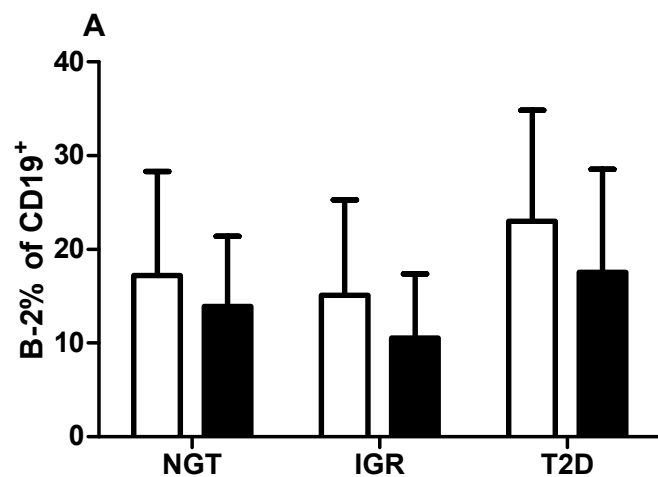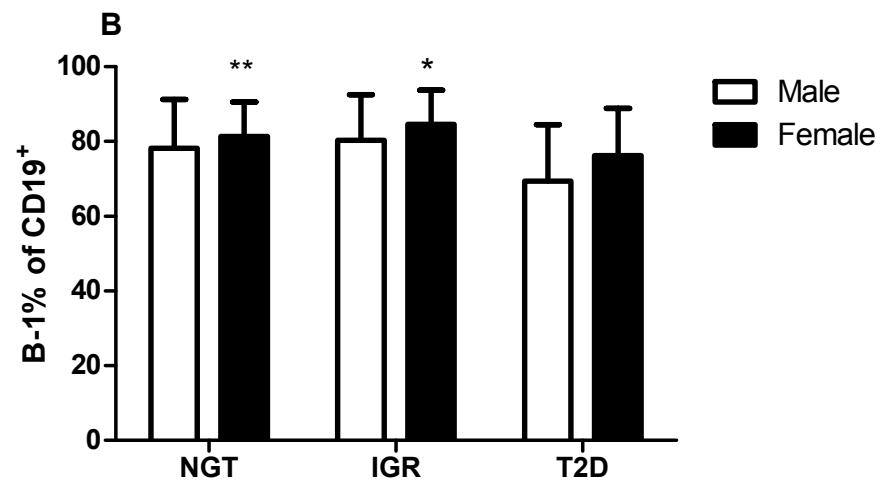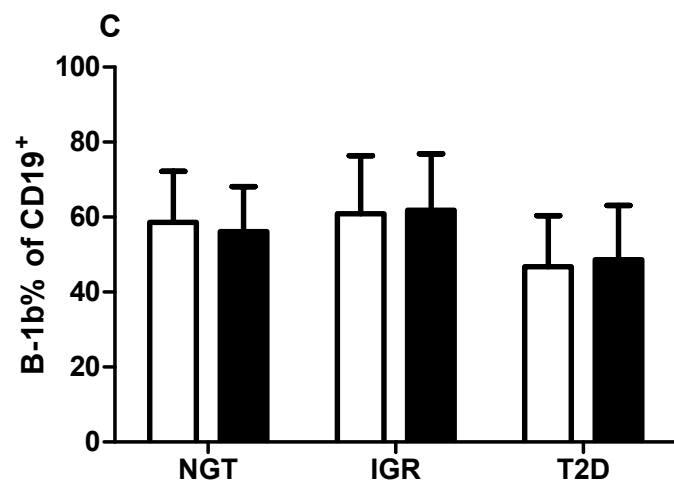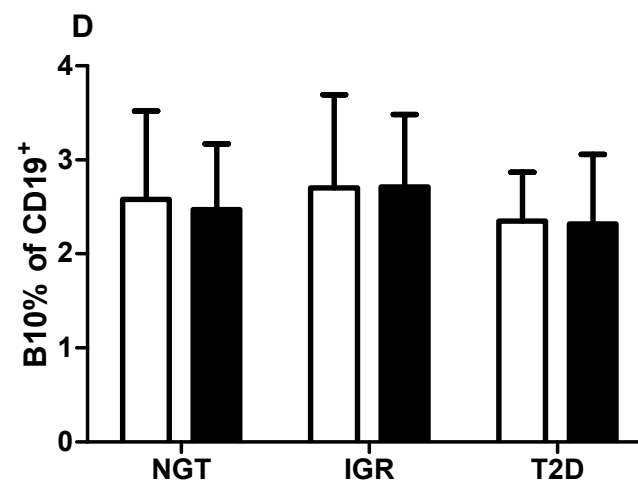

Supplement: Supplementary file 3 [file 5052812.f3.pdf]
